# *trans*-Tetra­carbonyl­bis­[tris­(4-fluoro­phen­yl)phosphane-κ*P*]chromium(0)

**DOI:** 10.1107/S1600536811033939

**Published:** 2011-08-27

**Authors:** M. N. Norlidah, F. M. Y. Hamdya, Omar Bin Shawkataly, Mohd Mustaqim Rosli, Hoong-Kun Fun

**Affiliations:** a Faculty of Industrial Science and Technology, Universiti Malaysia Pahang, Gambang 26300, Pahang, Malaysia; b Chemical Sciences Programme, School of Distance Education, Universiti Sains Malaysia, 11800 USM, Penang, Malaysia; c X-ray Crystallography Unit, School of Physics, Universiti Sains Malaysia, 11800 USM, Penang, Malaysia

## Abstract

In the title compound, [Cr(C_18_H_12_F_3_P)_2_(CO)_4_], the Cr atom is octa­hedrally coordinated by four carbonyl ligands and the two tertiary phosphanes that are *trans* to each other. The Cr atom and two carbonyl groups are on a twofold axis. The benzene rings attached to the phospho­rus atom make dihedral angles of 80.32 (5), 52.91 (5) and 83.80 (5)° with each other. In the crystal, C—H⋯O and C—H⋯F inter­molecular inter­actions form an infinite three-dimensional network.

## Related literature

For the crystal structures of phosphane complexes of carbonyl­chromium compounds, see: Preston *et al.* (1972 ▶); bin Shawkataly *et al.* (1996 ▶, 2009 ▶). For related structures, see: Brunet *et al.* (2002 ▶); Bennett *et al.* (2004 ▶). A search of the Cambridge Structural Database (Allen, 2002 ▶) reveals 113 complexes of carbonyl­chromium complexes with bis-phosphanes. For the stability of the temperature controller used in the data collection, see: Cosier & Glazer (1986 ▶). 
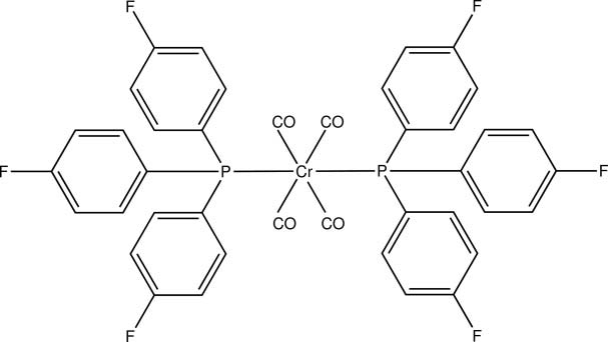


## Experimental

### 

#### Crystal data

[Cr(C_18_H_12_F_3_P)_2_(CO)_4_]
*M* *_r_* = 796.53
Monoclinic, 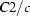

*a* = 11.9318 (8) Å
*b* = 18.0956 (8) Å
*c* = 15.8195 (8) Å
β = 92.740 (1)°
*V* = 3411.7 (3) Å^3^
*Z* = 4
Mo *K*α radiation
μ = 0.51 mm^−1^
*T* = 100 K
0.29 × 0.21 × 0.19 mm

#### Data collection

Bruker APEX Duo CCD area-detector diffractometer
Absorption correction: multi-scan (*SADABS*; Bruker, 2009 ▶) *T*_min_ = 0.869, *T*_max_ = 0.912
35263 measured reflections
5028 independent reflections
4627 reflections with *I* > 2σ(*I*)
*R*_int_ = 0.022

#### Refinement

*R*[*F* ^2^ > 2σ(*F* ^2^)] = 0.026
*wR*(*F* ^2^) = 0.074
*S* = 1.04
5028 reflections
242 parameters
H-atom parameters constrained
Δρ_max_ = 0.42 e Å^−3^
Δρ_min_ = −0.47 e Å^−3^

### 

Data collection: *APEX2* (Bruker, 2009 ▶); cell refinement: *SAINT* (Bruker, 2009 ▶); data reduction: *SAINT*; program(s) used to solve structure: *SHELXTL* (Sheldrick, 2008 ▶); program(s) used to refine structure: *SHELXTL*; molecular graphics: *SHELXTL*; software used to prepare material for publication: *SHELXTL* and *PLATON* (Spek, 2009 ▶).

## Supplementary Material

Crystal structure: contains datablock(s) I, global. DOI: 10.1107/S1600536811033939/ng5213sup1.cif

Structure factors: contains datablock(s) I. DOI: 10.1107/S1600536811033939/ng5213Isup2.hkl

Additional supplementary materials:  crystallographic information; 3D view; checkCIF report

## Figures and Tables

**Table 1:** Selected bond lengths (Å)

Cr1—C37	1.8808 (17)
Cr1—C38	1.8925 (11)
Cr1—C39	1.8989 (15)
Cr1—P1	2.3331 (3)

Symmetry code: (i) 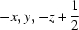.

**Table 2:** Hydrogen-bond geometry (Å, °)

*D*—H⋯*A*	*D*—H	H⋯*A*	*D*⋯*A*	*D*—H⋯*A*
C4—H4*A*⋯O1^ii^	0.93	2.55	3.4602 (16)	165
C8—H8*A*⋯F1^iii^	0.93	2.48	3.3830 (16)	165
C14—H14*A*⋯F3^iv^	0.93	2.46	3.3561 (14)	161

Symmetry codes: (ii) 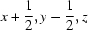; (iii) 
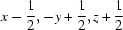
; (iv) 
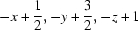
.

**Fig. 1.**
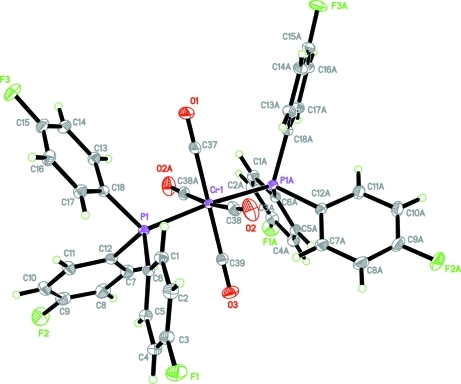
The molecular structure, showing 50% probability displacement ellipsoids. Hydrogen atoms are shown as spheres of arbitrary radius.

**Fig. 2.**
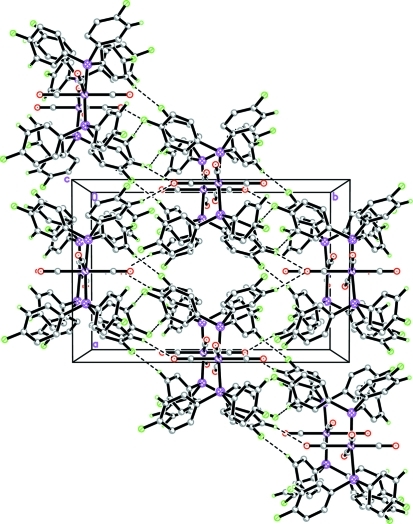
The crystal packing of (I). Dashed lines indicate hydrogen bonds. H atoms not involved in the hydrogen bond interactions have been omitted for clarity.

## supplementary crystallographic information

### Comment

The bonding characteristics of metal carbonyls with a phosphane ligand in phosphane-substituted metal carbonyls are interesting. Several crystal structures of phosphane substituted group 6 carbonyls with *trans* coordination of phosphane are reported (Brunet *et al.* 2002; Bennett *et al.*, 2004).

In the title compound, the Cr—P bond lengths, with an average value of 2.3331 (3) Å (Table 1) are relatively short in spite of the presence of the bulky phosphane ligand compared to the average values of 2.3656 (16)Å in the complex *trans*-Cr(CO)_4_(PPh_3_)_2_ (Bennett *et al.* 2004).

The Cr1, O1, O3, C37 C39 atoms lie on a twofold axis. The three benzene rings attached to the phosphorus atom make dihedral angles of 80.32 (5)° (between C1—C6 & C7—C12), 52.91 (5)° (between C1—C6 & C13—C18) and 83.80 (5)° (between C7—C12 & C13—C18) with each other.

In the crystal, the molecules are interconnected by intermolecular C4—H4A···O1^ii^, C8—H8A···F1^iii^ and C14—H14A···F3^iv^ interactions (Table 2) to form an infinite three-dimensional network (Fig. 2).

### Experimental

All manipulations were performed under a dry, oxygen-free nitrogen atmosphere using standard Schlenk techniques. All solvents were dried over sodium under dry oxygen free nitrogen. Chromium hexacarbonyl (200 mg, 0.909 mmol) and tris(4-flurophenyl)phosphane (301.8 mg, 0.9542 mmol) in 30 ml of pet ether (100–130°C) was refluxed for 12 h. Suitable single crystals were obtained by solvent-solvent diffusion in a mixture of dichloromethane/methanol.

### Refinement

All hydrogen atoms were positioned geometrically and refined using a riding model with C—H = 0.93Å and *U*_iso_(H) = 1.2*U*_eq_(C).

### Crystal data

**Table:**

[Cr(C_18_H_12_F_3_P)_2_(CO)_4_]	*F*(000) = 1616
*M**_r_* = 796.53	*D*_x_ = 1.551 Mg m^−^^3^
Monoclinic, *C*2/*c*	Mo *K*α radiation, λ = 0.71073 Å
Hall symbol: -C 2yc	Cell parameters from 9233 reflections
*a* = 11.9318 (8) Å	θ = 2.4–30.1°
*b* = 18.0956 (8) Å	µ = 0.51 mm^−^^1^
*c* = 15.8195 (8) Å	*T* = 100 K
β = 92.740 (1)°	Block, colourless
*V* = 3411.7 (3) Å^3^	0.29 × 0.21 × 0.19 mm
*Z* = 4	

### Data collection

**Table:**

Bruker APEX Duo CCD area-detector diffractometer	5028 independent reflections
Radiation source: fine-focus sealed tube	4627 reflections with *I* > 2σ(*I*)
graphite	*R*_int_ = 0.022
φ and ω scans	θ_max_ = 30.1°, θ_min_ = 2.1°
Absorption correction: multi-scan (*SADABS*; Bruker, 2009)	*h* = −16→16
*T*_min_ = 0.869, *T*_max_ = 0.912	*k* = −25→25
35263 measured reflections	*l* = −22→22

### Refinement

**Table:**

Refinement on *F*^2^	Primary atom site location: structure-invariant direct methods
Least-squares matrix: full	Secondary atom site location: difference Fourier map
*R*[*F*^2^ > 2σ(*F*^2^)] = 0.026	Hydrogen site location: inferred from neighbouring sites
*wR*(*F*^2^) = 0.074	H-atom parameters constrained
*S* = 1.04	*w* = 1/[σ^2^(*F*_o_^2^) + (0.035*P*)^2^ + 3.7272*P*] where *P* = (*F*_o_^2^ + 2*F*_c_^2^)/3
5028 reflections	(Δ/σ)_max_ = 0.001
242 parameters	Δρ_max_ = 0.42 e Å^−^^3^
0 restraints	Δρ_min_ = −0.47 e Å^−^^3^

### Special details

**Table:**

Experimental. The crystal was placed in the cold stream of an Oxford Cryosystems Cobra open-flow nitrogen cryostat (Cosier & Glazer, 1986) operating at 100.0 (1) K.
Geometry. All e.s.d.'s (except the e.s.d. in the dihedral angle between two l.s. planes) are estimated using the full covariance matrix. The cell e.s.d.'s are taken into account individually in the estimation of e.s.d.'s in distances, angles and torsion angles; correlations between e.s.d.'s in cell parameters are only used when they are defined by crystal symmetry. An approximate (isotropic) treatment of cell e.s.d.'s is used for estimating e.s.d.'s involving l.s. planes.
Refinement. Refinement of *F*^2^ against ALL reflections. The weighted *R*-factor *wR* and goodness of fit *S* are based on *F*^2^, conventional *R*-factors *R* are based on *F*, with *F* set to zero for negative *F*^2^. The threshold expression of *F*^2^ > σ(*F*^2^) is used only for calculating *R*-factors(gt) *etc*. and is not relevant to the choice of reflections for refinement. *R*-factors based on *F*^2^ are statistically about twice as large as those based on *F*, and *R*- factors based on ALL data will be even larger.

### Fractional atomic coordinates and isotropic or equivalent isotropic displacement parameters (Å^2^)

**Table:**

	*x*	*y*	*z*	*U*_iso_*/*U*_eq_	
Cr1	0.0000	0.470721 (12)	0.2500	0.01127 (6)	
P1	0.18184 (2)	0.464307 (14)	0.310682 (16)	0.01170 (6)	
F1	0.47047 (7)	0.30568 (5)	0.07445 (5)	0.03078 (18)	
F2	0.27164 (8)	0.30623 (5)	0.63765 (5)	0.0368 (2)	
F3	0.37981 (7)	0.75534 (4)	0.41277 (5)	0.02655 (16)	
O1	0.0000	0.63797 (7)	0.2500	0.0450 (4)	
O2	0.08864 (8)	0.48305 (6)	0.07365 (6)	0.0300 (2)	
O3	0.0000	0.30234 (6)	0.2500	0.0249 (2)	
C1	0.31342 (9)	0.45249 (6)	0.16868 (7)	0.01626 (19)	
H1A	0.2919	0.5010	0.1573	0.020*	
C2	0.37850 (9)	0.41494 (7)	0.11182 (7)	0.0195 (2)	
H2A	0.4017	0.4379	0.0630	0.023*	
C3	0.40757 (9)	0.34262 (7)	0.13002 (7)	0.0207 (2)	
C4	0.37508 (10)	0.30617 (6)	0.20114 (7)	0.0207 (2)	
H4A	0.3955	0.2572	0.2111	0.025*	
C5	0.31090 (9)	0.34444 (6)	0.25788 (7)	0.01666 (19)	
H5A	0.2883	0.3208	0.3064	0.020*	
C6	0.27996 (8)	0.41807 (6)	0.24279 (6)	0.01374 (18)	
C7	0.12666 (10)	0.36724 (6)	0.44384 (7)	0.0193 (2)	
H7A	0.0583	0.3604	0.4142	0.023*	
C8	0.14776 (11)	0.33051 (7)	0.52065 (8)	0.0247 (2)	
H8A	0.0940	0.2998	0.5428	0.030*	
C9	0.25007 (12)	0.34096 (6)	0.56260 (7)	0.0242 (2)	
C10	0.33278 (10)	0.38535 (7)	0.53176 (7)	0.0231 (2)	
H10A	0.4018	0.3906	0.5611	0.028*	
C11	0.31014 (9)	0.42195 (6)	0.45570 (7)	0.0191 (2)	
H11A	0.3647	0.4523	0.4340	0.023*	
C12	0.20668 (9)	0.41398 (6)	0.41100 (6)	0.01477 (18)	
C13	0.19260 (9)	0.59837 (6)	0.39426 (7)	0.01655 (19)	
H13A	0.1238	0.5831	0.4133	0.020*	
C14	0.23616 (9)	0.66625 (6)	0.42015 (7)	0.0186 (2)	
H14A	0.1979	0.6963	0.4567	0.022*	
C15	0.33815 (10)	0.68791 (6)	0.39001 (7)	0.0185 (2)	
C16	0.39903 (9)	0.64423 (6)	0.33799 (7)	0.0189 (2)	
H16A	0.4679	0.6600	0.3195	0.023*	
C17	0.35499 (9)	0.57552 (6)	0.31349 (7)	0.01725 (19)	
H17A	0.3957	0.5448	0.2792	0.021*	
C18	0.25037 (8)	0.55231 (5)	0.33991 (6)	0.01340 (17)	
C37	0.0000	0.57466 (9)	0.2500	0.0235 (3)	
C38	0.05647 (9)	0.47660 (6)	0.14043 (7)	0.0185 (2)	
C39	0.0000	0.36578 (8)	0.2500	0.0154 (3)	

### Atomic displacement parameters (Å^2^)

**Table:**

	*U*^11^	*U*^22^	*U*^33^	*U*^12^	*U*^13^	*U*^23^
Cr1	0.01065 (10)	0.00928 (10)	0.01385 (11)	0.000	0.00032 (7)	0.000
P1	0.01137 (11)	0.01041 (11)	0.01334 (12)	0.00025 (8)	0.00078 (8)	0.00089 (8)
F1	0.0299 (4)	0.0317 (4)	0.0315 (4)	0.0092 (3)	0.0101 (3)	−0.0112 (3)
F2	0.0589 (6)	0.0308 (4)	0.0198 (4)	−0.0033 (4)	−0.0080 (3)	0.0124 (3)
F3	0.0354 (4)	0.0177 (3)	0.0266 (4)	−0.0114 (3)	0.0022 (3)	−0.0056 (3)
O1	0.0433 (8)	0.0133 (6)	0.0749 (12)	0.000	−0.0335 (8)	0.000
O2	0.0223 (4)	0.0483 (6)	0.0196 (4)	−0.0018 (4)	0.0024 (3)	0.0089 (4)
O3	0.0365 (7)	0.0132 (5)	0.0250 (6)	0.000	0.0024 (5)	0.000
C1	0.0154 (4)	0.0155 (4)	0.0180 (5)	0.0012 (4)	0.0019 (4)	0.0001 (4)
C2	0.0173 (5)	0.0231 (5)	0.0185 (5)	0.0001 (4)	0.0042 (4)	−0.0012 (4)
C3	0.0165 (5)	0.0238 (5)	0.0221 (5)	0.0047 (4)	0.0020 (4)	−0.0085 (4)
C4	0.0213 (5)	0.0159 (5)	0.0245 (5)	0.0055 (4)	−0.0024 (4)	−0.0040 (4)
C5	0.0180 (5)	0.0142 (4)	0.0177 (4)	0.0018 (4)	−0.0011 (4)	−0.0002 (4)
C6	0.0121 (4)	0.0134 (4)	0.0156 (4)	0.0010 (3)	−0.0002 (3)	−0.0009 (3)
C7	0.0234 (5)	0.0168 (5)	0.0176 (5)	−0.0029 (4)	0.0008 (4)	0.0021 (4)
C8	0.0360 (6)	0.0184 (5)	0.0199 (5)	−0.0051 (5)	0.0023 (5)	0.0050 (4)
C9	0.0404 (7)	0.0168 (5)	0.0152 (5)	0.0029 (5)	−0.0016 (4)	0.0041 (4)
C10	0.0265 (5)	0.0227 (5)	0.0196 (5)	0.0039 (4)	−0.0050 (4)	0.0024 (4)
C11	0.0185 (5)	0.0204 (5)	0.0183 (5)	0.0009 (4)	−0.0007 (4)	0.0028 (4)
C12	0.0172 (4)	0.0126 (4)	0.0145 (4)	0.0017 (3)	0.0011 (3)	0.0010 (3)
C13	0.0157 (4)	0.0167 (5)	0.0174 (4)	−0.0012 (4)	0.0017 (3)	−0.0009 (4)
C14	0.0212 (5)	0.0167 (5)	0.0180 (5)	−0.0004 (4)	0.0012 (4)	−0.0038 (4)
C15	0.0244 (5)	0.0140 (4)	0.0168 (5)	−0.0054 (4)	−0.0022 (4)	−0.0004 (4)
C16	0.0184 (5)	0.0199 (5)	0.0185 (5)	−0.0062 (4)	0.0012 (4)	0.0004 (4)
C17	0.0157 (5)	0.0168 (5)	0.0194 (5)	−0.0020 (4)	0.0028 (4)	−0.0015 (4)
C18	0.0135 (4)	0.0121 (4)	0.0145 (4)	−0.0006 (3)	−0.0005 (3)	0.0008 (3)
C37	0.0188 (7)	0.0153 (7)	0.0350 (9)	0.000	−0.0117 (6)	0.000
C38	0.0138 (4)	0.0214 (5)	0.0200 (5)	−0.0004 (4)	−0.0013 (4)	0.0044 (4)
C39	0.0167 (6)	0.0159 (6)	0.0138 (6)	0.000	0.0013 (5)	0.000

### Geometric parameters (Å, °)

**Table:**

Cr1—C37	1.8808 (17)	C5—C6	1.4001 (14)
Cr1—C38^i^	1.8924 (11)	C5—H5A	0.9300
Cr1—C38	1.8925 (11)	C7—C12	1.3944 (15)
Cr1—C39	1.8989 (15)	C7—C8	1.3972 (15)
Cr1—P1^i^	2.3331 (3)	C7—H7A	0.9300
Cr1—P1	2.3331 (3)	C8—C9	1.3744 (19)
P1—C6	1.8288 (10)	C8—H8A	0.9300
P1—C18	1.8391 (10)	C9—C10	1.3796 (18)
P1—C12	1.8414 (10)	C10—C11	1.3886 (15)
F1—C3	1.3587 (12)	C10—H10A	0.9300
F2—C9	1.3570 (13)	C11—C12	1.4005 (15)
F3—C15	1.3595 (12)	C11—H11A	0.9300
O1—C37	1.146 (2)	C13—C14	1.3880 (15)
O2—C38	1.1471 (15)	C13—C18	1.4020 (14)
O3—C39	1.1481 (19)	C13—H13A	0.9300
C1—C2	1.3931 (15)	C14—C15	1.3846 (16)
C1—C6	1.4023 (14)	C14—H14A	0.9300
C1—H1A	0.9300	C15—C16	1.3737 (16)
C2—C3	1.3806 (16)	C16—C17	1.3973 (15)
C2—H2A	0.9300	C16—H16A	0.9300
C3—C4	1.3758 (17)	C17—C18	1.3998 (14)
C4—C5	1.3918 (15)	C17—H17A	0.9300
C4—H4A	0.9300		
			
C37—Cr1—C38^i^	86.78 (3)	C12—C7—C8	120.96 (11)
C37—Cr1—C38	86.78 (3)	C12—C7—H7A	119.5
C38^i^—Cr1—C38	173.56 (7)	C8—C7—H7A	119.5
C37—Cr1—C39	180.0	C9—C8—C7	118.26 (11)
C38^i^—Cr1—C39	93.22 (3)	C9—C8—H8A	120.9
C38—Cr1—C39	93.22 (3)	C7—C8—H8A	120.9
C37—Cr1—P1^i^	92.850 (8)	F2—C9—C8	119.05 (11)
C38^i^—Cr1—P1^i^	90.85 (3)	F2—C9—C10	118.04 (11)
C38—Cr1—P1^i^	89.47 (3)	C8—C9—C10	122.90 (11)
C39—Cr1—P1^i^	87.150 (8)	C9—C10—C11	118.12 (11)
C37—Cr1—P1	92.851 (8)	C9—C10—H10A	120.9
C38^i^—Cr1—P1	89.47 (3)	C11—C10—H10A	120.9
C38—Cr1—P1	90.85 (3)	C10—C11—C12	121.24 (11)
C39—Cr1—P1	87.149 (8)	C10—C11—H11A	119.4
P1^i^—Cr1—P1	174.299 (16)	C12—C11—H11A	119.4
C6—P1—C18	104.74 (5)	C7—C12—C11	118.50 (10)
C6—P1—C12	101.46 (5)	C7—C12—P1	122.43 (8)
C18—P1—C12	99.22 (5)	C11—C12—P1	119.07 (8)
C6—P1—Cr1	112.95 (3)	C14—C13—C18	121.25 (10)
C18—P1—Cr1	117.00 (3)	C14—C13—H13A	119.4
C12—P1—Cr1	119.17 (3)	C18—C13—H13A	119.4
C2—C1—C6	120.85 (10)	C15—C14—C13	118.16 (10)
C2—C1—H1A	119.6	C15—C14—H14A	120.9
C6—C1—H1A	119.6	C13—C14—H14A	120.9
C3—C2—C1	118.03 (10)	F3—C15—C16	118.55 (10)
C3—C2—H2A	121.0	F3—C15—C14	118.63 (10)
C1—C2—H2A	121.0	C16—C15—C14	122.81 (10)
F1—C3—C4	118.64 (10)	C15—C16—C17	118.40 (10)
F1—C3—C2	118.19 (11)	C15—C16—H16A	120.8
C4—C3—C2	123.17 (10)	C17—C16—H16A	120.8
C3—C4—C5	118.30 (10)	C16—C17—C18	120.86 (10)
C3—C4—H4A	120.9	C16—C17—H17A	119.6
C5—C4—H4A	120.9	C18—C17—H17A	119.6
C4—C5—C6	120.81 (10)	C17—C18—C13	118.45 (9)
C4—C5—H5A	119.6	C17—C18—P1	125.23 (8)
C6—C5—H5A	119.6	C13—C18—P1	116.31 (8)
C5—C6—C1	118.82 (9)	O1—C37—Cr1	180.0
C5—C6—P1	120.41 (8)	O2—C38—Cr1	177.10 (11)
C1—C6—P1	120.24 (8)	O3—C39—Cr1	180.0
			
C37—Cr1—P1—C6	−118.45 (4)	C7—C8—C9—C10	−0.71 (19)
C38^i^—Cr1—P1—C6	154.80 (5)	F2—C9—C10—C11	−178.90 (11)
C38—Cr1—P1—C6	−31.63 (5)	C8—C9—C10—C11	1.24 (19)
C39—Cr1—P1—C6	61.55 (4)	C9—C10—C11—C12	−0.34 (18)
C37—Cr1—P1—C18	3.27 (4)	C8—C7—C12—C11	1.58 (17)
C38^i^—Cr1—P1—C18	−83.48 (5)	C8—C7—C12—P1	−179.28 (9)
C38—Cr1—P1—C18	90.09 (5)	C10—C11—C12—C7	−1.03 (17)
C39—Cr1—P1—C18	−176.73 (4)	C10—C11—C12—P1	179.80 (9)
C37—Cr1—P1—C12	122.64 (4)	C6—P1—C12—C7	−111.54 (9)
C38^i^—Cr1—P1—C12	35.89 (5)	C18—P1—C12—C7	141.25 (9)
C38—Cr1—P1—C12	−150.55 (5)	Cr1—P1—C12—C7	13.13 (10)
C39—Cr1—P1—C12	−57.36 (4)	C6—P1—C12—C11	67.60 (9)
C6—C1—C2—C3	0.94 (16)	C18—P1—C12—C11	−39.61 (9)
C1—C2—C3—F1	179.61 (10)	Cr1—P1—C12—C11	−167.73 (7)
C1—C2—C3—C4	0.20 (17)	C18—C13—C14—C15	0.74 (16)
F1—C3—C4—C5	179.83 (10)	C13—C14—C15—F3	177.88 (10)
C2—C3—C4—C5	−0.76 (18)	C13—C14—C15—C16	−2.03 (17)
C3—C4—C5—C6	0.18 (16)	F3—C15—C16—C17	−178.85 (10)
C4—C5—C6—C1	0.92 (16)	C14—C15—C16—C17	1.06 (17)
C4—C5—C6—P1	172.56 (8)	C15—C16—C17—C18	1.22 (16)
C2—C1—C6—C5	−1.49 (16)	C16—C17—C18—C13	−2.42 (16)
C2—C1—C6—P1	−173.15 (8)	C16—C17—C18—P1	178.79 (8)
C18—P1—C6—C5	130.53 (8)	C14—C13—C18—C17	1.42 (16)
C12—P1—C6—C5	27.68 (9)	C14—C13—C18—P1	−179.68 (8)
Cr1—P1—C6—C5	−101.07 (8)	C6—P1—C18—C17	0.15 (10)
C18—P1—C6—C1	−57.95 (9)	C12—P1—C18—C17	104.68 (9)
C12—P1—C6—C1	−160.80 (8)	Cr1—P1—C18—C17	−125.76 (8)
Cr1—P1—C6—C1	70.45 (9)	C6—P1—C18—C13	−178.66 (8)
C12—C7—C8—C9	−0.74 (18)	C12—P1—C18—C13	−74.13 (9)
C7—C8—C9—F2	179.43 (11)	Cr1—P1—C18—C13	55.42 (9)

Symmetry codes: (i) −*x*, *y*, −*z*+1/2.

### Hydrogen-bond geometry (Å, °)

**Table:**

*D*—H···*A*	*D*—H	H···*A*	*D*···*A*	*D*—H···*A*
C4—H4A···O1^ii^	0.93	2.55	3.4602 (16)	165.
C8—H8A···F1^iii^	0.93	2.48	3.3830 (16)	165.
C14—H14A···F3^iv^	0.93	2.46	3.3561 (14)	161.

Symmetry codes: (ii) *x*+1/2, *y*−1/2, *z*; (iii) *x*−1/2, −*y*+1/2, *z*+1/2; (iv) −*x*+1/2, −*y*+3/2, −*z*+1.
